# Uniform carbon reserve dynamics along the vertical light gradient in mature tree crowns

**DOI:** 10.1093/treephys/tpae005

**Published:** 2024-01-10

**Authors:** Cedric Zahnd, Miro Zehnder, Matthias Arend, Ansgar Kahmen, Günter Hoch

**Affiliations:** Department of Environmental Sciences—Botany, University of Basel, Schönbeinstrasse 6, 4056 Basel, Switzerland; School of Biological Sciences, University of Utah, 257 South 1400 East, Salt Lake City, UT 84112, USA; Department of Environmental Sciences—Botany, University of Basel, Schönbeinstrasse 6, 4056 Basel, Switzerland; Department of Environmental Sciences—Botany, University of Basel, Schönbeinstrasse 6, 4056 Basel, Switzerland; Department of Geobotany, University of Trier, Universitätsring 15, 54296 Trier, Germany; Department of Environmental Sciences—Botany, University of Basel, Schönbeinstrasse 6, 4056 Basel, Switzerland; Department of Environmental Sciences—Botany, University of Basel, Schönbeinstrasse 6, 4056 Basel, Switzerland

**Keywords:** canopy gradient, carbon storage, non-structural carbohydrates, seasonal dynamics, temperate trees

## Abstract

Understanding the within-tree variability of non-structural carbohydrates (NSC) is crucial for interpreting point measurements and calculating whole-tree carbon balances. Yet, little is known about how the vertical light gradient within tree crowns influences branch NSC concentrations and dynamics. We measured NSC concentrations, irradiance and key leaf traits in uppermost, sun-exposed and lowest, shaded branches in the crowns of mature, temperate trees from nine species with high temporal resolution throughout one growing season. Measurements from two additional years allowed us to test the generality of our findings amongst climatically contrasting years. Despite the vertical light gradient, we found very similar seasonal NSC dynamics and concentrations between sun and shade branches in most species. This can at least partially be explained by acclimations in specific leaf area and photosynthetic leaf traits compensating the different light availability between the top and bottom canopy. Only in the ring-porous species *Quercus petraea* x *robur *and *Fraxinus*  *excelsior * was starch refilling after budbreak slower in lower branches. End-of-season NSC concentrations were similar between canopy positions and amongst observation years. Only *Fagus* sylvatica** had 40 and 29% lower starch concentrations by the end of the extremely dry year 2020, relative to the other 2 years. We show that NSC measured anywhere in a tree crown is often representative of the whole crown. Overall, our results suggest that carbon reserve dynamics in trees are largely insensitive to both microclimatic gradients and inter-annual climatic variation, and only deviate under severe carbon deficits, as was presumably the case with *Fagus* in our study.

## Introduction

Non-structural carbohydrates (NSC), consisting of starch and water-soluble sugars (hereafter referred to as sugars), constitute the major carbon (C) reserve compounds of trees (Chapin et al. 1990, Kozlowski 1992, Hoch et al. 2003). As such, NSC play a central role in keeping up the vital functions of trees both under normal conditions and after disturbances, like droughts or herbivore attacks (Hartmann et al. 2013, Lobo et al. 2013, O’Brien et al. 2014, Wiley 2020). Non-structural carbohydrates are accordingly an important component for tree and ecosystem models (McDowell et al. 2013, Lobo et al. 2013, Dietze et al. 2014). Since trees are large and modular organisms, understanding the within-tree variability of NSC and its temporal dynamics is crucial to properly interpret spatial and temporal point measurements and to use them in whole-tree C balance models. Non-structural carbohydrate concentrations differ widely amongst different tissues (i.e., bark, wood, leaves) and compartments (i.e., branches, stems, roots) of trees (Barbaroux et al. 2003, Hoch et al. 2003, Martínez-Vilalta et al. 2016, Gao et al. 2022). However, little is known about the variability of NSC within individual tree crowns. Indeed, particularly studies on seasonal NSC fluctuations are almost exclusively based on data measured in upper, sun-exposed branches (e.g., Sauter and van Cleve 1994, Schädel et al. 2009, Klein et al. 2016). Because of the vertical layering of foliage in tree crowns, leaves experience different microclimatic conditions, particularly in light availability, depending on their vertical position within the crown (Eliáš et al. 1989, Mariscal et al. 2004, Hertel et al. 2011). Typically, upper, high-irradiance leaves have a higher photosynthetic capacity (Lewis et al. 2000, Koike et al. 2001, Niinemets 2007). Since individual branches in tree crowns operate fairly autonomously (e.g., Sprugel et al. 1991), this may further influence the concentration and dynamics of branch NSC along the canopy light gradient.

Non-structural carbohydrates are the major intermediary pool between C assimilation and all plant processes that require assimilated C (e.g., respiration and growth) (Kozlowski 1992). Whenever photo-assimilation is not sufficient, NSC are the primary source for organic C (Fischer and Höll 1991, Newell et al. 2002, Smith and Stitt 2007). Therefore, NSC concentrations should in theory be proportional to the relative C source–sink balance of trees (Hoch 2015). Throughout a year, temporal imbalances between carbon assimilation and demand lead to distinct seasonal fluctuations in NSC, particularly in leaves and young branches (Kozlowski 1992, Hoch et al. 2003, Martínez-Vilalta et al. 2016). In temperate deciduous species, branch wood starch levels drop abruptly with budbreak and are subsequently refilled once the leaves become carbon autonomous (Schädel et al. 2009, Klein et al. 2016). Evergreen conifers on the other hand accumulate starch in the older needles and branch wood throughout early spring until budbreak, after which starch reserves decline again as they are used up for building new tissues (Hoch et al. 2003, Schädel et al. 2009). In contrast to starch, low molecular weight sugars, at least in temperate trees, tend to vary less seasonally (Schädel et al. 2009, Klein et al. 2016), and never fully deplete in living tissues (Martínez-Vilalta et al. 2016, Wiley et al. 2017, Weber et al. 2018).

**Figure 1:**
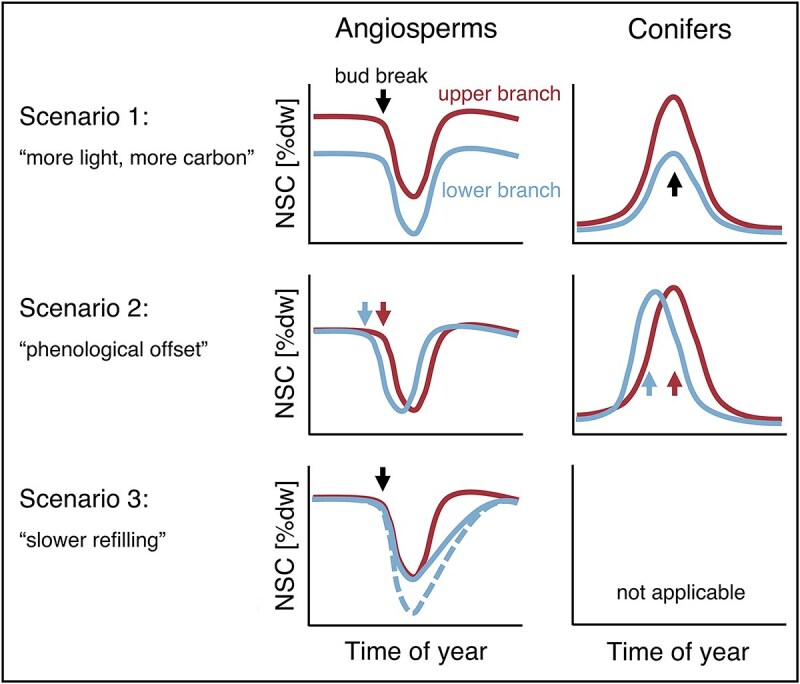
Conceptual figure of how the seasonal NSC dynamics of upper and lower crown branches could differ. Scenario 1: branches with lower light availability also have lower NSC concentrations, but the temporal dynamic is the same. Scenario 2: vertical offsets in the leaf and branch growth phenology lead to a temporally shifted NSC dynamic between upper and lower branches. Scenario 3: because of the lower light availability, lower branches use up relatively more NSC reserves during budbreak and/or take longer to refill reserves, but eventually reach similar NSC levels as the upper branches. Because conifers have much time in late winter and spring to build up reserves, it is unlikely that such delays in storage build-up would occur in lower branches of the conifers. Scenario 4, no difference in the NSC dynamics, is not shown in this figure.

As C assimilation rates, leaf morphology and sometimes phenology all change along the vertical light gradient within individual tree crowns (Ellsworth and Reich 1993, Niinemets 2007, Yoshimura 2013), so does the ratio of C source and sink activities and their temporal dynamics. Both the absolute NSC concentrations and their seasonal dynamics should therefore differ between sun-exposed and shaded branches. Assuming NSC largely reflects the difference between photo-assimilation and any non-NSC C sinks for now, several mutually non-exclusive scenarios can be formulated (Figure 1): First, because of the higher light availability and thus higher assimilation, upper branches may have higher NSC concentrations throughout the season (Figure 1, Scenario 1). Second, NSC in branches from different crown positions may also differ in their temporal dynamics throughout the year. As leaf phenology has a major impact on the seasonal starch dynamics, vertical variation in phenology (Koike et al. 2001, Yoshimura 2013) may cause temporal offsets of the seasonal NSC dynamics between sun and shade branches (Figure 1, Scenario 2). Third, in the absence of phenological variation, young leaves in the lower canopy of deciduous trees are getting shaded by upper foliage right from budbreak. Lower canopy branches may thus have to use up relatively more of their stored carbon for growing new tissues and may subsequently take longer to refill their stores (Figure 1, Scenario 3). Finally, NSC dynamics may not differ at all, either because physiological and morphological acclimations at the branch level are completely compensating the shade-effect on C assimilation, because C assimilates are redistributed within the canopy to compensate differences of the C-balance of individual branches, or because NSC reserves in branches are intrinsically maintained at specific tissue concentrations independent of the actual C source–sink balance of the individual branches.

Amongst the few studies looking at within-crown NSC patterns, some found differences between sun and shade branches (Meletiou-Christou et al. 1994, Warren and Adams 2001, Poorter et al. 2006), whereas others reported similar NSC concentrations within temperate (Li et al. 2001, 2009, Barbaroux et al. 2003, Schoonmaker et al. 2021) and tropical tree crowns (Würth et al. 2005, Martin et al. 2020, Signori-Müller et al. 2022). Existing data on within-crown NSC gradients are, however, sparse and often conflicting. Whilst it suggests that NSC decreases with increasingly shaded conditions at least in some cases, generalizations are currently hard to draw. To that end, we measured NSC concentrations in upper, sun-exposed and lower, shaded branches of nine common temperate tree species, including contrasting leaf habits (evergreen conifers and deciduous broadleaved species) and wood anatomies (conifers, ring- and diffuse-porous angiosperms). Utilizing the Swiss Canopy Crane II (SCC II) facility, we measured NSC concentrations throughout an entire season along with leaf phenology and the local light environment. We hypothesized that more sun-exposed branches have higher NSC concentrations and that lower, shaded branches use up more NSC during budbreak and/or take longer to refill reserves afterwards. To test the generality of our findings across climatically contrasting years, we also measured branch NSC in mid-summer and by the end of the season in two additional years.

## Materials and methods

### Site description

This study was conducted in a mature, temperate mixed forest at the SCCII research site (47.439 N, 7.776 E). At 550 m.a.s.l., the site is located within the foothills of the Jura Mountains, about 20 km south-east of Basel, Switzerland. Mean annual temperature and precipitation from 1991 to 2020 were 9.6 °C and 972 mm, respectively, based on data from the closest MeteoSwiss climate station in Rünenberg. The 1.68-ha research site contains 458 trees with a diameter at breast height (DBH) ≥10 cm. Of 14 tree species present, European beech and Norway spruce are the most common ones. The study forest is a relatively open and structurally diverse stand with a mean leaf area index of 2.2 and a basal area of 24.6 m^2^ ha^−1^. At the centre of the site, a 50-m tall canopy crane with a 50-m jib allowed access to the forest canopy via a manned gondola.

### Sampling design and in situ light measurements

The nine species studied here were the ring-porous broadleaved *Quercus petraea × robur* and *Fraxinus excelsior* L., the diffuse-porous broadleaved *Fagus sylvatica* L., *Acer pseudoplatanus* L., *Carpinus betulus* L. and *Sorbus torminalis* Crantz, and the evergreen conifers *Abies alba* Mill.*, Picea abies* Karst. and *Pinus sylvestris* L. All *Quercus* trees at the site are hybrids of different degrees between *Q. petraea* Liebl. and *Q. robur* L. (F. Guggerli, unpublished data), but treated as one species here. In the following, the species will be referred to by their genus names only. In all, 35 healthy, mature trees, with crowns reaching into the upper canopy were selected for this study (two to five trees per species, see Table 1). In each tree, one branch (ca 3 cm diameter) in the uppermost crown and one in the lowest, shaded crown were permanently marked. All phenological measurements were taken on these branches. On 15 of those trees (see Table 1 for the *N* of each species), light loggers (HOBO Pendant UA-002-64, Onset Computer Corp., Bourne, MA, USA) were installed on the top and bottom marked branches to measure light conditions on a 15-min interval from March 2019 to November 2021. Samples for the NSC concentration analysis were always collected from branches close to the permanently marked ones.

**Table 1:** Information on the sampled trees by species: mean DBH; mean and range of the heights of the upper and lower sampling locations (*H*_Upper_ and *H*_Lower_); number of trees sampled for NSC (*N*_NSC_); number of sampling timepoints in 2020 (*N*_Dates 20_); number of trees with light sensors installed on the upper and lower branches (*N*_Light_).

Species	Type	DBH (cm)	*H*_Upper_ (m) mean (min, max)	*H*_Lower_ (m) mean (min, max)	*N*_NSC_	*N*_Dates 20_	*N*_Light_
*Quercus*	Deciduous, ring-porous	55.4	30.6 (27.6, 31.9)	19.1 (13.7, 22.1)	5	11	2
*Fraxinus*	Deciduous, ring-porous	38.2	30.3 (27.7, 32.3)	18.0 (14.2, 21.4)	3	11	1
*Fagus*	Deciduous, diffuse-porous	49.6	29.1 (27.6, 30.9)	13.4 (11.0, 15.1)	5	13	4
*Acer*	Deciduous, diffuse-porous	46.5	30.4 (28.8, 31.5)	23.5 (22.2, 25.4)	3	11	1
*Carpinus*	Deciduous, diffuse-porous	29.1	22.8 (21.3, 24.4)	14.3 (10.1, 17.6)	3^3^	11	1
*Sorbus*	Deciduous, diffuse-porous	37.7	24.1 (21.3, 27.0)	13.7 (13.3, 14.1)	2	12	-
*Abies*	Evergreen conifer	43.9	32.6 (31.8, 34.9)	18.1 (15.8, 19.6)	4	10	2
*Picea*	Evergreen conifer	53.4	33.1 (31.9, 34.4)	18.8 (17.8, 20.7)	5^1^	12	2
*Pinus*	Evergreen conifer	47.9	32.5 (30.6, 34.4)	24.7 (21.7, 28.7)	5^2^	11	2

^1^One of these trees fell during a storm in February 2020 and was replaced by another tree.

^2^One of these trees was replaced after 2019, as it escaped the reach from the crane.

^3^One of these trees died in spring 2021 from snow breakage and was replaced for the 2021 measurements.

Note that in December 2019, only *Fagus*, *Quercus*, *Picea* and *Pinus* (three trees each) were sampled.

### Leaf phenology and growth

The leaf phenology data used here were previously described in detail by Zahnd et al. (2023). In brief, leaf development, from budbreak to fully unfolded leaves, was recorded one to two times per week on all the permanently marked branches. For this study, the time from 10% budbreak to 90% of leaves fully unfolded was defined as the period of leaf development. Stem increment growth was recorded at breast height on the stem of the same trees using manual band dendrometers (Meter Group AG, Munich, Germany). Dendrometers were read out weekly throughout the year 2020. The approximate period of stem increment growth was found visually from the seasonal dendrometer curves.

### Non-structural carbohydrate sampling

Samples for NSC concentration analysis were collected at high frequency throughout the year 2020. Starting in January for the conifers and in early March for the broadleaved trees, samples were taken in up to weekly intervals during the period of leaf development, and in approximately monthly intervals for the remaining season, with an additional sampling on 30 November, after all broadleaved trees had shed their leaves. Additionally, samples were taken twice in the years 2019 and 2021: once in mid-summer (28 June 2019 and 15 June 2021) and once at the end of the growing season (5 December 2019 and 2 December 2021). Because of time constraints, only *Fagus*, *Quercus*, *Picea* and *Pinus* (three trees each) were sampled in December 2019. To compare all 3 years at similar timepoints, samples taken on 16 June (conifers) and 14 July (angiosperms) were used as the mid-summer samples in 2020, and those collected on 30 November 2020 for the end of the season of that year.

At each sampling date, between 10 a.m. and 3 p.m., a 3–5-year-old shoot was collected close to all the permanently marked branches in the top and bottom crowns (within ca 1 m). Because of the very large and few branches of *Fraxinus*, 1-year-old shoots were sampled from that species. Upon collection, the bark was removed from the branch wood samples and 1-year-old needles were separated. Samples were immediately heat shocked in a microwave. The samples were then dried for 72 h at 85 °C, ground to a fine powder using a mixer mill (MM 400, Retsch GmbH, Haan, Germany) and stored in plastic tubes on desiccant until chemical analysis was performed.

### Non-structural carbohydrate analysis

We analyzed NSC concentrations in the branch wood and 1-year-old needles (conifers only). Because leaves of deciduous trees are not a longer-term storage compartment for trees, we did not analyze the seasonal NSC dynamics in leaves of broadleaved species in detail for this study; however, we measured leaf NSC concentrations in top and bottom canopy branches of all broadleaved species at one date in summer 2020 (between 4 May and 5 June, depending on species). NSC analysis was performed following the enzymatic-photometric methods detailed in Landhäusser et al. (2018). In brief, low molecular sugars were extracted from the dried samples with an 80% ethanol solution at 90 °C. Sucrose was then converted to glucose and fructose using invertase (1 mg mL^−1^) at room temperature, and fructose was subsequently converted to glucose by isomerase. The resulting total amount of glucose (representing to the sum of glucose, fructose and sucrose in the original sample) was then quantified photometrically at 340 nm on a multiplate-photometer (HR 700, Hamilton, Reno, NE, USA) after conversion of glucose to gluconate-6-P using hexokinase (glucose assay reagent, Sigma Aldrich, St Louis, MO, USA). The starch remaining in the pellet after the ethanol extraction was digested with α-amylase (600 U mL^−1^) at 85 °C and subsequently with amyloglucosidase (12 U mL^−1^) at 55 °C. The total amount of free glucose after starch degradation was determined photometrically as described above. Absorption values were converted to concentrations (% dry mass) using a calibration curve measured with a standard glucose solution. Sucrose, fructose and starch solutions, as well as two different plant powders (orchard leaves and cereal grains), were included in each analysis run to ensure correct enzyme activities and reproducibility of the analyses. All chemicals and enzymes were purchased from Sigma Aldrich (Merck KGaA, Darmstadt, Germany).

### Specific leaf area and photosynthetic light response

For determining the specific leaf area (SLA), leaves and 1-year-old needles were collected on 30 September 2021 from small branches near the permanently marked ones. Fresh leaf area of broadleaved species was measured with a planimeter (LI-3100C Area Meter, LI-COR Biosciences GmbH, Bad Homburg, Germany). The conifer needles were scanned using a flat-bed scanner and the one-sided projected leaf area was determined using a dedicated digital image analysis tool (github.com/dabasler/LeafAreaExtraction). Leaves were subsequently dried for 72 h at 85 °C and then weighed.

For each species, photosynthetic light response curves (LRC) were measured on one sun and one shade leaf in one tree and on two dates in 2021: once in June (14 and 16) and once in July (21 and 22), always between 10 a.m. and 3 p.m., resulting in two LRCs per species and canopy position (except for *Fraxinus*, where all four curves were measured in July). Environmental conditions on these dates were close to ideal for photosynthesis, with average midday temperatures of 24.7 and 21.9 °C, and relative humidity (RH) of 43.9 and 65.0%, on the measurement dates in June and July, respectively. Volumetric soil moisture content was around 42% (measured at 10 cm soil depth in a clay-rich leptosol). For each LRC, net assimilation rate (*A*_N_) was measured at 11 light intensities from 1800 to 0 μmol m^−2^ s^−1^ using a LI-6800 Portable Photosynthesis System (LI-COR Biosciences GmbH, Bad Homburg, Germany). Temperature and RH in the leaf chamber were kept constant at ca 25 °C and 60%, respectively. For the conifers, *A*_N_ was corrected for the actual needle area in the chamber by measuring the one-sided projected needle area as described above. Each LRC was fitted with nine different, commonly used functions as summarized by Lobo et al. (2013). Based on residual standard errors, the Ye model (Ye 2007) fit best or close to best in all cases. Therefore, the following function for *A*_N_ was used for all LRCs:


$$ {A}_{\mathrm{N}}={\mathrm{\phi}}_{\left({I}_0-{I}_{\mathrm{c}}\right)}\times \frac{1-\beta \times I}{1+\gamma \times I}\times \left(I-{I}_{\mathrm{c}}\right) $$

where *A*_N_ is the net photosynthesis rate (μmol CO_2_ m^2^s^−1^), *I* is the photosynthetic photon flux density (PPFD; μmol photons m^2^s^−1^), *I*_c_ is the light compensation point (μmol photons m^2^s^−1^), ${\mathrm{\phi}}_{\left({I}_0-{I}_{\mathrm{comp}}\right)}$ is the quantum yield between *I*_0_ and *I*_c_ (μmol CO_2_ μmol photon^−1^) and *β* and *γ* are dimensionless adjusting factors. Light-saturated net assimilation rates (*A*_sat_) and dark respiration rates (*R*_d_) were determined from these curves as described in Ye (2007).

### Data analysis

Light readings from the HOBO loggers were converted from lux to photosynthetically active PPFD (μmol m^−2^ s^−1^) using the empirically established relationship PPFD = lux/120 (Möhl et al. 2020). Quarter-hourly readings were then summed up to give diurnal light integrals (DLI; mmol m^−2^ day^−1^). For each of the 15 trees (see Table 1), relative irradiance was calculated as the DLI in the lower crown expressed as percentage of the DLI measured in the top crown.

To test differences in the timing of leaf phenology between crown positions, we analyzed the midpoints between 10% budbreak and 90% fully unfolded leaves with a linear mixed effects model, using crown position, species and their interaction as fixed effects and tree ID as random intercept. We further compared that midpoint, averaged across crown positions, to the stem growth onset, using a linear mixed effects model with species as additional fixed effect and tree ID as random intercept. Timing differences were tested for each species individually using post hoc *t*-tests.

Starch and soluble sugar concentrations were always analyzed separately, as were the two tissues (wood and 1-year-old needles in the conifer species). The seasonal starch and sugar dynamics in 2020 were analyzed using linear mixed effects models with crown position, the interaction term ‘species × date’ (where date is a categorical variable) and their threefold interaction as fixed effects and a tree ID as random intercept. As NSC sampling was adapted according to each species’ specific phenology, not all species were sampled on the same dates. Therefore, the species and date effects could not be tested individually, and for that reason only the ‘species × date’ interaction term, but not the individual terms, was included in the model. For each species and sampling date, the difference between crown positions (upper and lower) was further tested individually using post hoc *t*-tests.

To test differences between crown positions in mid-summer and at the end of the season over the 3 years 2019–21, separate linear mixed effects models were fitted for each species and tissue, each with either starch or sugar as response variable and with crown position (top or bottom), sampling timepoint (as factor) and their interaction as fixed effects. A tree ID was included as random intercept to account for repeated measurements. Based on these models, differences between crown positions were tested for each timepoint using post hoc *t*-tests. To further test whether starch or sugar concentrations at the end of the season differed between years, post hoc *t*-tests were performed on the according marginal means (averaged across canopy positions) of those same models.

Specific leaf area, *I*_c_, *A*_sat_ and *R*_d_ were each analyzed with linear mixed effect models using crown position, species and their interaction as fixed effects and tree ID as random intercept. To ensure normality of residuals, SLA was log-transformed. Post hoc *t*-tests were used to test differences between crown positions in each species. Note that for *I*_c_, *A*_sat_ and *R*_d_, we only had two replicates (*n* = 2), statistical significances of these parameters should therefore be viewed with caution.

All data analysis was done in the R statistical environment (R core team 2022). Mixed effects models were formulated using the package ‘lme4’ (Bates et al. 2015) and post hoc tests were performed using the package ‘emmeans’ (Lenth 2022).

## Results

### Canopy light gradient

**Figure 2:**
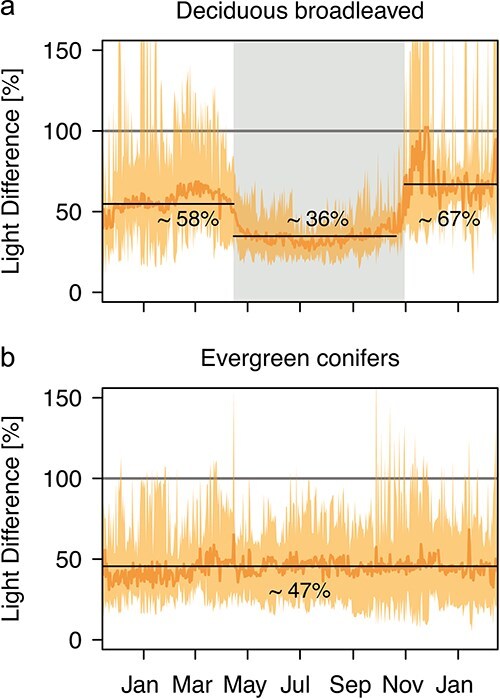
Light difference between upper and lower branches. Diurnal light integral (DLI) in the lower branches relative to the upper branches for (a) deciduous broadleaved species and (b) evergreen conifers throughout 2020. Mean (dark orange) and maximum and minimum (orange shading) relative DLI across all trees is shown. Black lines and numbers indicate the overall means and the gray area in (a) shows the average vegetation season across all broadleaved species. Underlying data was originally published in Zahnd et al. (2023).

There was a distinct light gradient within the tree crowns all year round, although with a seasonal pattern in the broadleaved species: from late autumn to early spring, lower branches received on average between 58.1% (± 5.93 SE) and 67.2% (± 6.72 SE) of the light reaching the upper branches. After leaves developed, this fraction was significantly reduced to ca 36.2% (± 2.42 SE) during the growing season (paired *t*-test: *t* = 4.80, df = 8, *P* = 0.001; Figure 2). In contrast, the mean relative light availability in the lower branches of evergreen conifers (DLI of lower relative to upper branches) was not significantly influenced by neighboring deciduous trees and stayed quite constant throughout the year at ca 46.9% (± 4.64 SE; paired *t*-test: *t* = −0.86, df = 5, *P* = 0.426), although the variability amongst conifer individuals was slightly reduced during the growing season (Figure 2). The relative light availability tended to differ further between the deeper-crowned conifers *Abies* and *Picea* (34.2% ± 0.27 and 39.4% ± 11.05, respectively) and the more shallow-crowned *Pinus* (67.9% ± 13.71).

In both broadleaved species and conifers alike, sun and shade branches showed significantly different frequency distributions of light intensities (Table S1 available as Supplementary data at *Tree Physiology* Online): shade branches had a higher proportion of PPFD values below 100 μmol m^−2^ s^−1^, but lower proportions above 300 μmol m^−2^ s^−1^ (Figure S1 available as Supplementary data at *Tree Physiology* Online). Furthermore, the proportion of light intensities below the net-photosynthetic light compensation points (*I*_c_) was similar for sun and shade branches in ring-porous species (17.9 and 18.6%, respectively, Welch two sample *t*_2.4_ = −0.49, *P* = 0.663) and conifers (30.4 and 33.2%, *t*_5.7_ = −0.76, *P* = 0.477), whereas shade branches of diffuse-porous species had a slightly but significantly higher frequency of light intensities above *I*_c_ (17.5 and 14.9%, *t*_10_ = 2.49, *P* = 0.032).

### Leaf and growth phenology

**Figure 3:**
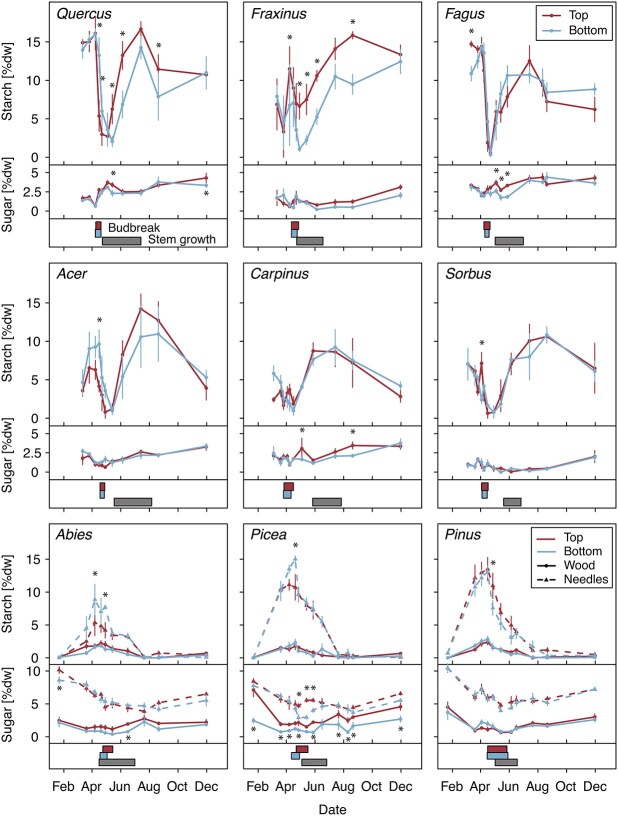
Seasonal NSC dynamics of the nine species. Starch (upper curves) and sugar (lower curves) concentrations throughout the year 2020 in the wood (solid lines) and needles (dashed, conifers only) of upper crown (red) and lower crown (blue) branches. Boxes at the bottom show leaf development (10% budbreak to 90% of leaves fully unfolded, see Table S4, available as Supplementary data at *Tree Physiology* Online, for precise dates) of lower (blue) and upper (red) crown branches and the period of stem increment growth (gray). Note that the leaf phenology data were taken from Zahnd et al. (2023). Stars indicate significant differences in starch or sugar concentrations between canopy positions based on post hoc *t*-tests (^*^P ≤ 0.05).

Spring leaf phenology, from budbreak to fully unfolded leaves, occurred almost simultaneously in upper and lower branches of most species (Tables S2 and S4 available as Supplementary data at *Tree Physiology* Online). Only two of the conifers, *Picea* and *Abies*, showed significant within-crown differences, with the midpoint of leaf phenology occurring about 13 and 9 days earlier in lower compared with upper branches of *Picea* and *Abies*, respectively (Figure 3, Table S3 available as Supplementary data at *Tree Physiology* Online, see also Zahnd et al. 2023). Additionally, lower leaves of *Carpinus* developed slightly but significantly faster than upper leaves (3.5 days, Table S3 available as Supplementary data at *Tree Physiology* Online). Although the timing of leaf flushing and the onset of secondary stem growth differed significantly in all species but *Fraxinus*, *Picea* and *Pinus* (Table S3 available as Supplementary data at *Tree Physiology* Online), differences also tended to be small in the second ring-porous species (*Quercus*) and the third conifer species (*Abies*, Figure 3). On the other hand, there was a particularly pronounced and significant lag between leaf flushing and stem growth onset in the early flushing *Carpinus* and *Sorbus* (Figure 3, Table S3 available as Supplementary data at *Tree Physiology* Online).

### Overall seasonal NSC dynamics

All deciduous broadleaved species showed the expected seasonal patterns of NSC concentrations in branch wood, with a depletion of starch around spring budbreak followed by a fast refilling of starch reserves during the first half of the growing season (Figure 3). This general pattern was most consistent in *Fagus* and *Quercus*, where starch concentrations were high at around 13–15% dry weight (d.w.) in March, then dropped with the onset of budbreak abruptly to close to 0% d.w. and started to increase again upon full unfolding of the leaves. After reaching maximum concentrations in July, starch gradually declined again towards autumn and winter. Whilst we cannot statistically test qualitative differences in the seasonal dynamics amongst species, the remaining broadleaved species appear to show the same general pattern, albeit with some notable differences: The second ring-porous species besides *Quercus*, *Fraxinus*, showed an initial drop of starch concentrations in late March, followed by a more pronounced drop around budbreak in April (Figure 3), whereas the early flowering *Carpinus* already had relatively low starch concentrations on the first measurement point in early March (ca 5–7% d.w., Figure 3). Finally, starch in *Acer* branches increased in March before leaf-out, which occurred in April in this species (Figure 3). In all broadleaved species, refilling of branch wood starch reserves occurred either before or at the same time as stem increment growth (Figure 3). Sugar concentrations and their seasonal fluctuations in branches of broadleaved trees were generally lower than those of starch. Overall, sugar concentrations tended to be highest during winter and decreased to lower levels during spring and summer (Figure 3).

In contrast to the deciduous broadleaved species, starch concentrations in the evergreen conifers were lowest and close to 0% d.w. in mid-winter (January) in both branch wood and needles, increased during late winter and spring and reached a maximum at budbreak (Figure 3). Starch concentrations then gradually decreased again until autumn, staying at low levels for the rest of the year (Figure 3). This pattern was observed in needles and wood, although much stronger in the needles, which reached maximum concentrations of 5–15% d.w. at budbreak, whereas wood starch concentrations were always relatively low and never exceeded 4% d.w. throughout the season. Like in the broadleaved deciduous species, the seasonal fluctuations of sugars were much less pronounced than starch but showed the highest concentrations over winter. This pattern was particularly pronounced in the needles, where fluctuations were stronger than in the wood of both conifers and broadleaved species (Figure 3, Figure S2 available as Supplementary data at *Tree Physiology* Online).

### Crown position effects on seasonal NSC dynamics

Averaged over all species, there was no significant difference in starch concentrations between upper and lower branches throughout the year 2020 in either the wood or needles (Table 2). Indeed, seasonal dynamics were very similar in upper and lower branches of most species (Figure 3). Accordingly, the seasonal amplitudes (i.e., the difference between the seasonal maximum and minimum concentrations) of starch also did not differ significantly between upper and lower branches in most species (Figure S2, Table S5 available as Supplementary data at *Tree Physiology* Online). However, significant interaction effects of crown position with species and date on the starch concentration in both tissues suggest that concentrations did differ between crown positions in some species and at individual timepoints (Table 2). Although post hoc tests revealed occasional significant differences in most species, only a few showed clear patterns of differing seasonal dynamics between crown positions. First, the two ring-porous species (*Fraxinus* and *Quercus*) had higher starch concentrations in the upper branches for most of the season starting around budbreak (Figure 3). Whilst the starch concentration in upper branches of *Quercus* dropped more abruptly and subsequently refilled more quickly, starch concentrations in the upper branches of *Fraxinus* were generally ca 5% d.w. higher, but with similar temporal dynamics to the lower branches. In both species, however, starch in upper and lower branches converged again towards the end of the season, reaching very similar concentrations by the end of autumn (Figure 3). Second, in the needles of *Abies* and *Picea*, lower branches reached significantly higher peak starch concentrations at budbreak than upper branches (+3.5 and +4.3% d.w., respectively), but the concentrations of top and bottom branches quickly converged thereafter (Figure 3). Because of this, lower branches of *Picea* and *Abies* also had larger seasonal amplitudes of needle starch concentrations compared with upper branches, although only in *Abies* significantly so (*P* = 0.003, Figure S2 available as Supplementary data at *Tree Physiology* Online).

**Table 2:** Analysis of deviance table (type II Wald χ^2^ tests) of the starch and soluble sugar concentrations throughout the year 2020 in wood and 1-year-old needles (conifers only). Note that species and date effects could not be tested individually, as species were not always sampled on the same dates.

Response	Variable	χ^2^	df	*P*
Starch—Wood	Crown position	1.52	1	0.217^ns^
	Species:Date	1774.68	101	<0.001^*^^*^^*^
	Crown pos.:Species:Date	171.29	101	<0.001^*^^*^^*^
Starch—Needles	Crown position	0.78	1	0.378^ns^
	Species:Date	1602.11	32	<0.001^*^^*^^*^
	Crown pos.:Species:Date	48.35	32	0.032^*^
Sugar—Wood	Crown position	70.47	1	<0.001^*^^*^^*^
	Species:Date	1095.61	101	<0.001^*^^*^^*^
	Crown pos.:Species:Date	223.94	101	<0.001^*^^*^^*^
Sugar—Needles	Crown position	9.98	1	0.002^*^^*^
	Species:Date	611.03	32	<0.001^*^^*^^*^
	Crown pos.:Species:Date	45.67	32	0.056^ns^

^^*^^*^^*^^ *P* ≤ 0.001,

^^*^^*^^ *P* ≤ 0.01,

^^*^^ *P* ≤ 0.05,

^ns^ *P* > 0.05

In contrast to starch, sugar concentration overall differed significantly between canopy positions in both wood and needles (Table 2), although the threefold interaction of crown position with species and date was significant in the wood samples and approached significance in the needles (*P* = 0.056, Table 2). Individually, several species tended to have higher sugar concentrations in the upper branches for at least part of the season (Figure 3), which was most consistent in the *Picea* branch wood. Other species, particularly *Acer*, *Sorbus* and *Pinus* showed no such differences at all. Amplitudes of the seasonal sugar fluctuations were again very similar in upper and lower branches of most species (Figure S2 and Table S5 available as Supplementary data at *Tree Physiology* Online). Individually, only the upper branch wood samples of *Picea* had a significantly larger amplitude compared with lower branches (*P* < 0.001, Figure S2 available as Supplementary data at *Tree Physiology* Online), which was mainly driven by the about threefold higher sugar concentrations of top vs bottom branches in January (Figure 3).

### Interannual differences in branch NSC

**Figure 4:**
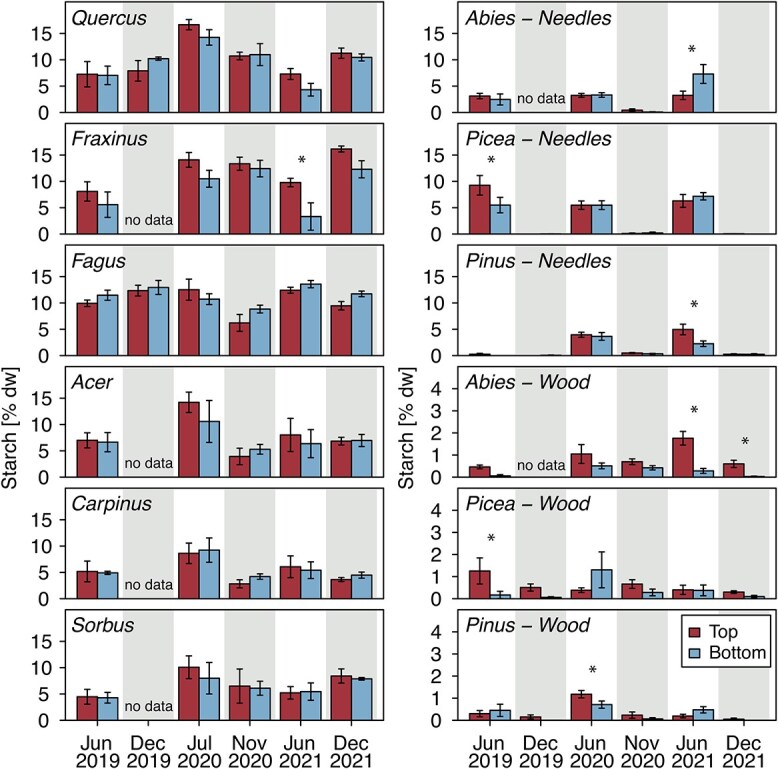
Starch concentrations in mid-summer and at the end of the season over 3 years. Starch concentrations in upper (red) and lower (blue) branch wood and needles as measured in mid-summer (white background) and at the end of the season (gray background) of the years 2019 to 2021. Significant differences between crown positions based on post hoc *t*-tests are indicated with stars (^*^P < 0.05). Note the different *y* axis scale of the three conifer branch wood plots (lower right).

The very similar starch concentrations in top and bottom canopy branches observed throughout the year 2020 generally held true in the other two observation years (2019 and 2021, Figure 4), but with a few exceptions: although in most cases not statistically significant, there was a consistent tendency for mid-summer starch concentrations to be higher in the upper than the lower branches of the ring-porous species (*Quercus* and *Fraxinus*). Additionally, mid-summer starch concentrations in both wood and needles of all three conifer species significantly differed between crown positions occasionally, but not with any discernible pattern (Figure 4). Differences in sugar concentrations between upper and lower branches were more common than in starch, but again varied amongst species and timepoints. Notably, whenever concentrations did differ, upper branches always had higher sugar concentrations than lower branches (Figure S3 available as Supplementary data at *Tree Physiology* Online).

The three study years were characterized by strongly different climatic conditions. With mean summer temperatures (June–August) of 19.5 and 18.5 °C, both 2019 and 2020 had above-average hot summers (1.7 and 0.7 K above the 30-year average, respectively). The years 2019 and particularly 2020 were also extremely dry years, with growing season climatic water balances (April–September) of −143.2 and −213.9 mm, respectively (−228.5 and −299.0 mm below 30-year average, respectively). The year 2021, in comparison, was a markedly cooler year (17.5 °C mean summer temperature, 0.3 K below the 30-year average), with a particularly wet growing season (+236.5-mm climatic water balance, 151.4 mm above 30-year average). Yet, both the end-of-season starch and sugar concentrations (averaged over both crown positions) did not differ amongst the years in most species (see Figure 4, Figure S3 and Table S6 available as Supplementary data at *Tree Physiology* Online). For starch, the only exception was *Fagus*, which showed significantly reduced starch concentrations in 2020 relative to 2019 and 2021 (−5.1 and −3.1% d.w., respectively, Table S6 available as Supplementary data at *Tree Physiology* Online).

### Specific leaf area and photosynthetic light response

**Figure 5:**
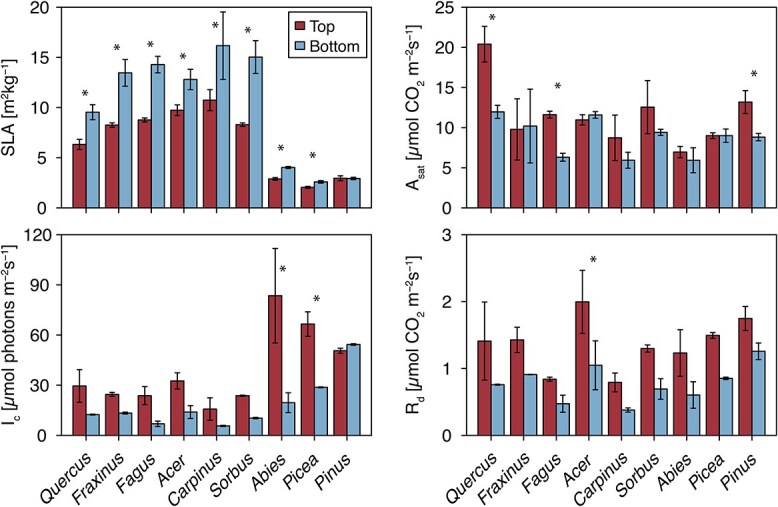
Leaf traits of sun and shade leaves. SLA (top left), light compensation point (*L*_c_, bottom left), light-saturated net assimilation rate (*A*_sat_, top right) and dark respiration rate (*R*_d_, bottom right) of leaves from the upper (red) and lower (blue) crowns of the nine species. *L*_c_, *A*_sat_ and *R*_d_ were derived from photosynthetic LRC. Significant differences between crown positions based on post hoc *t*-tests are indicated with stars (^*^P < 0.05). For SLA, the *N* per species is the same as given in Table 1 (*N*_NSC_). The three LRC parameters have *N* = 2, statistical significances should thus be treated with caution.

Shade leaves of eight species had significantly higher SLA than sun leaves, with differences ranging from 29.0% higher values of shade leaves in *Picea* to 81.7% in *Sorbus* (Figure 5, Table 3, Table S7 available as Supplementary data at *Tree Physiology* Online)*.* Only *Pinus* showed no significant difference in SLA between sun and shade leaves (0.8%, Figure 5). We found overall significant crown position effects in the light-saturated net assimilation rates (*A*_sat_), the net-photosynthetic light compensation points (*I*_c_) and the leaf dark respiration rates (*R*_d_), however, with significant species × crown position interactions in the case of *A*_sat_ and *I*_c_ (Table 3). Sun leaves tended to have higher *I*_c_ and *R*_d_ rates in most species, although individually only significantly so in *Abies* and *Picea* (*I*_c_) and *Acer* (*R*_d_, Figure 5, Table S7 available as Supplementary data at *Tree Physiology* Online). *A*_sat_ tended to be more similar between crown positions, with only *Quercus*, *Fagus* and *Pinus* showing significantly higher *A*_sat_ rates in sun leaves (Figure 5, Table S7 available as Supplementary data at *Tree Physiology* Online).

**Table 3:** Analysis of deviance table (type II Wald χ^2^ tests) of the SLA (log-transformed) and three parameters derived from photosynthetic LRC: light compensation point (*I*_c_), light-saturated net assimilation rate (*A*_sat_) and dark respiration rate (*R*_d_).

Response	Variable	χ^2^	df	*P*
SLA	Crown position	93.37	1	<0.001^*^^*^^*^
	Species	1445.94	8	<0.001^*^^*^^*^
	Crown position:Species	22.08	8	0.005^*^^*^
*I*_c_	Crown position	31.19	1	<0.001^*^^*^^*^
	Species	74.11	8	<0.001^*^^*^^*^
	Crown position:Species	24.92	8	0.002^*^^*^
*A*_sat_	Crown position	18.10	1	<0.001^*^^*^^*^
	Species	4.93	8	0.765^ns^
	Crown position:Species	20.70	8	0.008^*^^*^
*R*_d_	Crown position	30.25	1	<0.001^*^^*^^*^
	Species	11.96	8	0.153^ns^
	Crown position:Species	2.29	8	0.971^ns^

^^*^^*^^*^^ *P* ≤ 0.001,

^^*^^*^^ *P* ≤ 0.01,

^ns^ *P* > 0.05

## Discussion

In this study, we revealed that, within a given species, the seasonal dynamics and tissue concentrations of NSC were surprisingly similar between young branches from top and bottom locations of mature tree crowns. This finding was rather unexpected considering the stark vertical light gradient during the growing season. The similarity was especially strong for starch, except for the two investigated ring-porous species which showed a delayed refilling of starch in bottom branches (cf. Scenario 3 in Figure 1). These results were consistent across 3 years: particularly at the end of the growing seasons, all species had very similar starch concentrations in top and bottom branches and amongst the three observation years, despite different climatic conditions. Overall, our findings suggest that branch NSC concentrations and dynamics in trees are largely insensitive to both microclimatic and climatic variation, and only deviate under severe C deficit, as was, in our study, presumably the case with *Fagus* during the summer drought 2020.

### Seasonal starch dynamics of sun and shade branches

Because of the vertical light gradients characterizing mature tree crowns, we hypothesized that shade branches would have generally lower starch concentrations, use more of their starch reserves during leaf flushing and/or take longer to subsequently refill these reserves (Figure 1). Contrary to these expectations, the starch concentrations throughout 2020 were for all but the ring-porous species largely identical in upper, sun exposed branches and lower, shaded branches, both quantitatively and in terms of their temporal dynamics. This pattern also held true across the two additional measurement years. Whilst previous studies have reported similar NSC concentrations in upper and lower branches of tree crowns, these usually measured relatively young trees in open stands (Li et al. 2001, 2009, Barbaroux et al. 2003, Schoonmaker et al. 2021). It is therefore unlikely that those trees experienced strong vertical light gradients. In the current study, we found that even the up to 75% reduction of light availability did, in all but the two ring-porous species, largely not affect branch NSC concentrations. Though mature, our study site is relatively open (mean LAI of 2.2), and more closed stands can experience even stronger vertical light gradients (e.g., Hollinger 1996, Koike et al. 2001, Poorter et al. 2006). Hence, we cannot exclude that lowest branches in denser canopies with steeper light gradients would show reduced NSC concentrations. However, previous experimental studies with tree saplings exposed to deep shade (6% of full sunlight) also revealed no difference in wood NSC concentrations between shaded and unshaded saplings after a 3-year acclimation period (Weber et al. 2019). Based on our current observation and the previous experimental findings we do not expect to find very pronounced vertical NSC concentration gradients in even denser canopies than the one investigated here.

Interestingly, we found the most consistent differences between crown positions in the two investigated ring-porous species: in line with our hypothesized Scenario 3 (Figure 1), both *Quercus* and *Fraxinus* showed slower starch refilling after budbreak in lower branches, only reaching the starch concentrations of upper branches by the end of the season. Since leaves developed simultaneously across the tree crowns in both species (see also data in Zahnd et al. 2023), however, leaf phenology cannot be the cause of these observed differences. Instead, they might relate to the early onset of secondary wood formation in ring-porous temperate trees (Zweifel et al. 2006, Sass-Klaassen et al. 2011, Klein et al. 2016, Lavrič et al. 2017). In contrast to diffuse-porous species, ring-porous trees have to produce new vessels prior to leaf flushing in spring, because most of the large vessels in ring-porous wood cavitate over winter (Cochard and Tyree 1990, Bréda and Granier 1996). The high C demand for wood formation prior to budbreak is likely causing a stronger reliance of ring-porous trees on C reserves, particularly in the early season (Barbaroux and Bréda 2002). Gričar et al. (2017) additionally found that stem growth in oak precedes growth in branches by up to a month, which may suggest that lower branches also start growing before upper ones. The assumed higher demand for stored C, but also currently assimilated C, for wood growth during the early growing season might thus explain the delayed refilling of NSC reserves in lower branches of ring-porous species. In diffuse-porous species on the other hand, the secondary stem growth only sets in after most of the branch C reserves have been refilled.

We observed only one other notable instance of differing starch concentrations: for a brief period before budbreak in 2020, needles on lower branches of *Abies* and *Picea* reached higher starch concentrations than those in upper branches. Additionally, mid-summer needle starch and sugar concentrations differed between canopy positions in some species and years (Figure 4, Figure S3 available as Supplementary data at *Tree Physiology* Online). These differences are not consistent with any of our expected scenarios (Figure 1), and since effects went into both directions (upper branches had sometimes higher and sometimes lower concentrations), we cannot discern a clear effect of the light environment or phenology on these differences. Similarly, Warren and Adams (2001) reported higher NSC in the needles of sun vs shade needles of *Pinus pinaster* trees shortly before budbreak, but not by the end of the growing season, whereas Meletiou-Christou et al. (1994) found either higher, lower or similar starch concentrations in the lower compared with upper crown leaves of four evergreen sclerophyllous tree species, depending on the species and, to some extent, the season. Based on these observations, it appears that there is some as yet unexplained, and potentially stochastic, within-crown variability in the evergreen foliage NSC, particularly during the period of peak biological activity. Whilst we did not analyze NSC in leaves of broadleaved species in detail, measurements from one date in early summer 2020 revealed for most species no difference in starch and sugar concentrations between crown positions (Figure S4 and Table S8 available as Supplementary data at *Tree Physiology* Online).

### End of season NSC concentrations

Not only were the end of season starch concentrations largely identical between top and bottom branches, but they were also very similar for the three investigated years, despite the significantly different climatic conditions amongst years. Carbon reserves therefore seem largely insensitive to climatic variation across years, suggesting that C reserves in branch wood might be regulated to species-specific concentrations independent of the actual growing season conditions. The only clear deviation from that pattern was found in *Fagus*, where starch levels by the end of 2020 were significantly lower than in the other years (Table S6 available as Supplementary data at *Tree Physiology* Online). Additionally, in that particular year, starch tended to be lower in the upper branches of *Fagus*. The lower NSC concentrations in 2020 coincided with both an extreme drought, leading to premature leaf shedding in the upper canopy leaves of *Fagus*, as well as a large masting event for *Fagus*, with fruit production predominantly occurring in the upper canopy (pers. obs.). Since fruit production is primarily supported by current assimilates, which would be also needed to refill branch C stores (Hoch et al. 2013), this, in combination with the drought, may have caused the end-of-season reduction of starch in the xylem. Similar reductions in branch starch concentrations after droughts have been reported before (Arend et al. 2022), whereas others found no reduction of branch NSC concentrations in European beech even after repeated droughts (Hesse et al. 2021).

### The different patterns of starch and sugars

In contrast to the highly dynamic, phenology-driven seasonal changes in starch, low molecular sugar concentrations showed less seasonal fluctuations in most species. Whilst sugars were generally low throughout summer and higher in winter, they, compared with starch, were more constant throughout the season and never fully depleted. These findings agree with previous studies on seasonal NSC dynamics (Schädel et al. 2009, Klein et al. 2016, Martínez-Vilalta et al. 2016), and are likely caused by the multiple other physiological functions of sugars, as opposed to starch, which exclusively functions as a storage compound (Morgan 1984, Gibson 2005, Savage et al. 2016). Within the tree crowns, sugar concentrations were often similar between top and bottom branches, but notably, whenever they did differ, they were higher in sun branches. One possible reason for this might be increasing the cold resistance in upper, more exposed branches, since particularly strong differences in sugar concentrations can be observed in winter (Graham and Patterson 1982, Amundson et al. 1992, Ruelland et al. 2009). Another reason for higher sugar concentrations in upper branches, particularly in summer, may be osmotic adjustment to the increasing hydraulic constraints with increasing tree height (Merchant et al. 2006, Woodruff and Meinzer 2011, Venturas et al. 2017).

### Why are differences along the canopy light gradient not more pronounced?

Considering that the strong light gradient in mature tree canopies would suggest much higher C assimilation in sun compared with shade branches, the very similar starch dynamics and concentrations in upper and lower branches of most species were remarkable. Of course, well-known morphological and physiological acclimations allow leaves to optimize C assimilation to their specific light environment. For instance, leaf size, display angle, SLA, chlorophyll content and nitrogen allocation are all adjusted along the vertical light gradient (Koike et al. 2001, Niinemets 2007, Peltoniemi et al. 2012, Lobo et al. 2013, Bachofen et al. 2020). These adjustments typically result in higher maximum assimilation rates of high-irradiance leaves, but lower respiration and better low-light photosynthetic efficiency in shade leaves (Koike et al. 2001, Larcher 2003). However, whether such adjustments fully compensate for the given light gradient, resulting in a similar C source–sink balance of sun and shade branches, is difficult to tell.

In our case, shade leaves of most species indeed tended to have higher SLA, lower photosynthetic light compensation points (*I*_c_) and lower respiration rates (*R*_d_). On the other hand, saturated assimilation rates of sun leaves only exceeded that of shade leaves in three species, including one ring-porous, one diffuse-porous and one conifer species. Furthermore, whilst overall light availability was reduced for shade leaves across all species (Figures 2, Figure S1 available as Supplementary data at *Tree Physiology* Online), frequency of light intensities below *I*_c_ was similar for sun and shade branches in ring-porous species and conifers, and differed only slightly in diffuse-porous species. It follows that, whilst we see light gradients strong enough to induce at least some leaf acclimations in our trees, none of these acclimations can readily explain why there are differences in the starch concentrations of sun and shade branches in the ring-porous, but not the other species. Calculating seasonal C balances of sun and shade branches may help to disentangle the effects of light availability and leaf- and branch-level acclimations on the NSC concentrations. Indeed, previous studies have calculated significantly longer C amortization times for the production costs of shade compared with sun leaves, suggesting that the acclimations may not suffice to fully equalize the C balance along the vertical light gradient (Poorter et al. 2006, Cavatte et al. 2012).

Several other mechanisms could therefore contribute to explaining the similar NSC dynamics we observed. First, assimilates may be redistributed from the upper to the lower branches in order to equilibrize the NSC concentrations. Lacointe et al. (2004) showed that extensive redistribution of carbohydrates amongst branches is possible under severe experimentally induced C starvation, although under natural conditions this appears to be a rare and very limited process (Sprugel et al. 1991 and references therein; Volpe et al. 2008). Second, the similar NSC concentrations might be achieved through differential C allocation: in that case, shade branches would invest proportionally more of their assimilates into storage, whereas sun branches would export more assimilates towards other parts of the tree. Finally, the vertical light gradient also causes higher summer temperatures and vapor pressure deficits (VPD) in the upper crown, which limits photosynthesis of sun leaves and thus reduces the difference in net C uptake of sun and shade branches (Ruimy et al. 1995, Niinemets et al. 2004, Bachofen et al. 2020). Considering the very similar starch concentrations between top and bottom canopy branches in most species, it seems rather unlikely that they are caused primarily by such VPD canopy gradients. However, they might play a role for the observed differences in *Quercus* and *Fraxinus*, both species with deeper reaching roots and anisohydric stomatal control (e.g., Zweifel et al. 2009, Leuschner et al. 2019, Kahmen et al. 2022).

Morphological and physiological light acclimations likely reduced the difference in C source-sink balance of sun and shade branches at least to some extent. However, the almost complete absence of starch concentration differences along the vertical light gradient in many of the studied species and timepoints may also indicate a high priority for refilling C reserves over other C sink activities, with significant differences only occurring during periods of presumably very strong C deficit, like the early season wood formation in ring-porous trees or the restricted C assimilation in drought-prone upper branches of *Fagus* in 2020. Stable or even increasing NSC concentrations under C limiting circumstances have been shown experimentally (Lacointe et al. 2004, Wiley et al. 2017, Weber et al. 2019), indicating that C can indeed be invested in storage at the cost of growth or other C sink activities (Sala et al. 2012, Wiley and Helliker 2012, Huang et al. 2021). But whether this is also the case under natural within-crown shading will require an assessment of the seasonal branch-level C balance. Although the biochemical and genetic processes behind starch formation, remobilization and sugar transport are fundamentally well understood (e.g., Smith and Stitt 2007), it remains largely unknown how the distribution and mobilization of starch are controlled across the diverse parts of large trees. One possible explanation for the very similar NSC concentrations along the light gradient and across the climatically contrasting years is that, whenever possible, starch simply fills up to capacity. Whilst this hypothesis is supported by the close correlations sometimes found between starch concentrations and the fraction of parenchyma and living fibers in wood (Silva et al. 2014, Plavcová et al. 2016; but see Godfrey et al. 2020), it is not clear whether the storage capacity of branches itself changes along the vertical canopy gradient. Whatever the precise mechanisms, our results show that starch values, particularly towards the end of the growing season, measured anywhere in the crown are often representative of the entire crown and can be used for calculating whole-tree C models.

## Supplementary Material

Supplementary_tpae005

## Data Availability

Phenology and light data are available at https://doi.org/10.6084/m9.figshare.21952826. NSC data are available at https://doi.org/10.6084/m9.figshare.23559837.
