# Supplementary material for: Uniform carbon reserve dynamics along the vertical light gradient in mature tree crowns
Source: Tree Physiol. 2024 Jan 10;44(13):232–45. doi: 10.1093/treephys/tpae005 (PMC11898625; doi:10.1093/treephys/tpae005)
Supplement: Supplementary_tpae005 [file supplementary_tpae005.docx]

**Supplementary Material to “Uniform carbon reserve dynamics along the vertical light gradient in mature tree crowns”: Additional results.**

*1 Distribution of light intensities in upper and lower branches.*

**Fig. S1:** Counts of light intensities, in bins of 100 PPFD, measured in upper (red) and lower (blue) branches of broadleaved species (left panel) and conifers (right panel) throughout 2020. Only non-zero measurements were included. The proportion of different light intensities differed significantly between crown positions (see Table S1).

**Table S1:** Analysis of Deviance table (type II Wald χ^2^ tests) of the light intensity counts (in bins of 100). Data was analysed using a generalised linear mixed effects model with a log link (Poisson distribution). Bin number, crown position (upper vs. lower) and tree type (broadleaved vs. conifers) and all their interaction effects were used as fixed factors, with a tree ID as random intercept. Post-hoc z-ration tests showed significant crown position effects in all bins of both broadleaved and conifer trees.

| Response | Variable | χ^2^ | df | *P* |
| --- | --- | --- | --- | --- |
| Count of Light | Bin | 584470 | 10 | <0.001 *** |
| Intensities | Crown position | 47.95 | 1 | <0.001 *** |
|  | Type | 1.02 | 1 | 0.312 ^ns^ |
|  | Bin:Crown position | 44569 | 10 | <0.001 *** |
|  | Bin:Type | 872.91 | 10 | <0.001 *** |
|  | Crown position:Type | 314.27 | 1 | <0.001 *** |
|  | Bin: Crown position:Type | 1341.7 | 10 | <0.001 *** |

*** *P* ≤ 0.001; ** *P* ≤ 0.01; * *P* ≤ 0.05; ns *P* > 0.05

*2. Additional information on leaf and growth phenology. Note that leaf phenology data was originally published in Zahnd et al. (2023).*

**Table S2:** Analysis of Deviance table (type II Wald χ^2^ tests) of leaf and growth phenological parameters. ‘Leaf flushing’ refers to the comparison of the leaf phenological mid-point (between 10 % budbreak and 90 % fully unfolded) between crown positions and among species. ‘Leaf v. growth phenology’ refers to the comparison of that same mid-point, now averaged across crown positions, to the onset of stem increment growth. See main text Fig. 3.

| Response | Variable | χ^2^ | df | *P* |
| --- | --- | --- | --- | --- |
| Leaf flushing | Crown position | 50.63 | 1 | <0.001 *** |
|  | Species | 217.81 | 8 | <0.001 *** |
|  | Crown position:Species | 105.14 | 8 | <0.001 *** |
| Leaf v. growth | Leaf v. stem | 63.65 | 1 | <0.001 *** |
| phenology | Species | 68.41 | 8 | <0.001 *** |
|  | Leaf v. stem:Species | 205.58 | 8 | <0.001 *** |

*** *P* ≤ 0.001; ** *P* ≤ 0.01; * *P* ≤ 0.05; ns *P* > 0.05

**Table S3:** Species-wise comparison of leaf flushing dates between upper and lower branches, and of leaf flushing date vs onset of stem increment growth. Differences were tested with post-hoc *t*-tests on the models in Table S1.

| Response | Species | Contrast | Estimate ± SE | df | t ratio | *P* |
| --- | --- | --- | --- | --- | --- | --- |
| Leaf flushing | *Quercus* | bottom - top | -0.7 ± 1.28 | 27 | -0.55 | 0.590 |
|  | *Fraxinus* | bottom - top | -1.33 ± 1.66 | 27 | -0.8 | 0.428 |
|  | *Fagus* | bottom - top | -0.17 ± 1.17 | 27 | -0.14 | 0.888 |
|  | *Acer* | bottom - top | -0.5 ± 1.66 | 27 | -0.3 | 0.765 |
|  | *Carpinus* | bottom - top | -3.5 ± 1.66 | 27 | -2.11 | 0.044 * |
|  | *Sorbus* | bottom - top | -0.5 ± 2.03 | 27 | -0.25 | 0.807 |
|  | *Abies* | bottom - top | -9 ± 1.43 | 27 | -6.27 | < 0.001 *** |
|  | *Picea* | bottom - top | -13.5 ± 1.28 | 27 | -10.52 | < 0.001 *** |
|  | *Pinus* | bottom - top | 0.5 ± 1.28 | 27 | 0.39 | 0.700 |
| Leaf v. growth | *Quercus* | leaf – growth | -7.55 ± 3.05 | 23 | -2.48 | 0.021 * |
| phenology | *Fraxinus* | leaf – growth | -3.17 ± 3.94 | 23 | -0.8 | 0.429 |
|  | *Fagus* | leaf – growth | -16.7 ± 3.05 | 23 | -5.48 | < 0.001 *** |
|  | *Acer* | leaf – growth | -24.58 ± 3.94 | 23 | -6.24 | < 0.001 *** |
|  | *Carpinus* | leaf – growth | -48.63 ± 4.82 | 23 | -10.08 | < 0.001 *** |
|  | *Sorbus* | leaf – growth | -39.25 ± 4.82 | 23 | -8.14 | < 0.001 *** |
|  | *Abies* | leaf – growth | 15.37 ± 3.41 | 23 | 4.51 | < 0.001 *** |
|  | *Picea* | leaf – growth | -3.94 ± 3.41 | 23 | -1.15 | 0.260 |
|  | *Pinus* | leaf – growth | 6.69 ± 3.41 | 23 | 1.96 | 0.062 |

*** *P* ≤ 0.001; ** *P* ≤ 0.01; * *P* ≤ 0.05

**Table S4:** Mean date (± SD in days) of bud break, fully unfolded leaves, stem growth onset and stem growth cessation. Leaf phenology dates are given for the top and bottom crown separately. See main text Fig. 3. Leaf phenology data was originally published in Zahnd et al. (2023).

| Species | Crown pos. | Leaf phenology | | Stem growth phenology | |
| --- | --- | --- | --- | --- | --- |
|  |  | Bud break (± SD) | Unfolded (± SD) | Onset (± SD) | End (± SD) |
| *Quercus* | Top | 10. Apr (± 3.91) | 21. Apr (± 1.79) | 23. Apr (± 6.62) | 04. Aug (± 26.11) |
|  | Bottom | 09. Apr (± 3.78) | 21. Apr (± 1.79) |  |  |
| *Fraxinus* | Top | 13. Apr (± 2.31) | 28. Apr (± 5.69) | 23. Apr (± 6.24) | 28. Jun (± 15.01) |
|  | Bottom | 13. Apr (± 2.31) | 25. Apr (± 4.36) |  |  |
| *Fagus* | Top | 09. Apr (± 3.56) | 22. Apr (± 4.13) | 02. May (± 4.80) | 31. Jul (± 4.60) |
|  | Bottom | 11. Apr (± 2.37) | 20. Apr (± 3.61) |  |  |
| *Acer* | Top | 19. Apr (± 4.93) | 29. Apr (± 7.23) | 18. May (± 12.01) | 06. Aug (± 7.64) |
|  | Bottom | 19. Apr (± 5.20) | 28. Apr (± 7.81) |  |  |
| *Carpinus* | Top | 28. Mar (± 9.87) | 17. Apr (± 3.21) | 27. May (± 7.07) | 06. Aug (± 7.78) |
|  | Bottom | 27. Mar (± 10.97) | 12. Apr (± 2.00) |  |  |
| *Sorbus* | Top | 04. Apr (± 0.00) | 18. Apr (± 2.83) | 20. May (± 2.83) | 30. Jul (± 46.67) |
|  | Bottom | 05. Apr (± 1.41) | 16. Apr (± 0.00) |  |  |
| *Abies* | Top | 25. Apr (± 4.86) | 16. May (± 5.69) | 16. Apr (± 6.60) | 13. Jul (± 24.54) |
|  | Bottom | 19. Apr (± 5.19) | 04. May (± 8.06) |  |  |
| *Picea* | Top | 23. Apr (± 3.56) | 18. May (± 6.43) | 04. May (± 7.50) | 08. Aug (± 34.11) |
|  | Bottom | 13. Apr (± 5.18) | 30. Apr (± 7.58) |  |  |
| *Pinus* | Top | 17. Apr (± 2.59) | 28. May (± 2.24) | 02. May (± 3.00) | 29. Jun (± 8.04) |
|  | Bottom | 16. Apr (± 2.35) | 30. May (± 4.09) |  |  |

*3. Amplitudes of seasonal starch and sugar fluctuations*

**Fig. S2:** Amplitude of seasonal starch (upper panel) and sugar (lower panel) fluctuations in the 1-yo needles (left) and wood (right) of upper crown (red) and lower crown (blue) branches. Stars indicate significant differences between canopy positions based on post-hoc *t*-tests (* *P* ≤ 0.05; ** *P* ≤ 0.01; *** *P* ≤ 0.001).

**Table S5:** Analysis of Deviance table (type II tests) of the amplitude of seasonal starch and soluble sugar fluctuations in wood and 1-yo needles (conifers only).

| Response | Variable | χ^2^ | df | *P* |
| --- | --- | --- | --- | --- |
| Starch - Wood | Species | 420.34 | 8 | <0.001 *** |
|  | Crown position | 2.30 | 1 | 0.129 ^ns^ |
|  | Species: Crown position | 8.32 | 8 | 0.403 ^ns^ |
| Starch - Needles | Species | 36.57 | 2 | <0.001 *** |
|  | Crown position | 7.63 | 1 | 0.006 ** |
|  | Species: Crown position | 10.55 | 2 | 0.005 ** |
| Sugar - Wood | Species | 14.70 | 8 | 0.065 ^ns^ |
|  | Crown position | 6.75 | 1 | 0.009 ** |
|  | Species: Crown position | 16.80 | 8 | 0.032 * |
| Sugar - Needles | Species | 4.31 | 2 | 0.116 ^ns^ |
|  | Crown position | 0.19 | 1 | 0.663 ^ns^ |
|  | Species: Crown position | 3.88 | 2 | 0.144 ^ns^ |

*** *P* ≤ 0.001; ** *P* ≤ 0.01; * *P* ≤ 0.05; ns *P* > 0.05

*4. Sugar concentrations in upper and lower branches over three years*

**Fig. S3:** Sugar concentrations in mid-summer and at the end of the season over three years. Sugar concentrations in upper (red) and lower (blue) branch wood and needles as measured in mid-summer (white background) and at the end of the season (grey background) of the years 2019 to 2021. Significant differences between crown positions based on post-hoc *t*-tests are indicated with stars (* *P* < 0.05). Refer to Fig. 5 of the main text for the according starch concentrations.

*5. Interannual differences in branch starch and sugar concentrations*

**Table S6:** Differences in end-of-season starch and soluble sugar concentrations among the two or three years. Differences were tested with post-hoc *t*-tests on the 3-year models, averaging values over both canopy positions (top and bottom).

|  |  |  | Starch | | Sugar | |
| --- | --- | --- | --- | --- | --- | --- |
| Species | Tissue | Contrast | Diff. [% d.w] | *P* | Diff. [% d.w] | *P* |
| *Quercus* | Wood | 2019-2020 | -1.792 | 0.278 | 0.319 | 0.471 |
|  |  | 2020-2021 | 0.002 | 0.999 | -0.344 | 0.364 |
|  |  | 2019-2021 | -1.79 | 0.279 | -0.024 | 0.956 |
| *Fraxinus* | Wood | 2020-2021 | -1.333 | 0.365 | -0.33 | 0.310 |
| *Fagus* | Wood | 2019-2020 | 5.117 | <0.001 *** | -0.426 | 0.173 |
|  |  | 2020-2021 | -3.071 | 0.006 ** | -0.563 | 0.036 * |
|  |  | 2019-2021 | 2.046 | 0.099 | -0.989 | 0.003 ** |
| *Acer* | Wood | 2020-2021 | -2.294 | 0.208 | -0.974 | 0.002 ** |
| *Carpinus* | Wood | 2020-2021 | -0.711 | 0.542 | -0.409 | 0.192 |
| *Sorbus* | Wood | 2020-2021 | -1.851 | 0.072 | 0.165 | 0.663 |
| *Abies* | Wood | 2020-2021 | 0.246 | 0.203 | -0.024 | 0.932 |
|  | Needles | 2020-2021 | 0.257 | 0.697 | 1.552 | 0.019 * |
| *Picea* | Wood | 2019-2020 | -0.187 | 0.627 | -0.849 | 0.087 |
|  |  | 2020-2021 | 0.274 | 0.400 | 0.71 | 0.088 |
|  |  | 2019-2021 | 0.088 | 0.819 | -0.139 | 0.776 |
|  | Needles | 2019-2020 | 0.053 | 0.957 | 0.346 | 0.528 |
|  |  | 2020-2021 | 0.122 | 0.880 | 0.341 | 0.450 |
|  |  | 2019-2021 | 0.175 | 0.859 | 0.686 | 0.214 |
| *Pinus* | Wood | 2019-2020 | -0.059 | 0.701 | -0.092 | 0.789 |
|  |  | 2020-2021 | 0.123 | 0.340 | 0.117 | 0.686 |
|  |  | 2019-2021 | 0.064 | 0.679 | 0.025 | 0.942 |
|  | Needles | 2019-2020 | -0.396 | 0.428 | -0.198 | 0.588 |
|  |  | 2020-2021 | 0.17 | 0.661 | 0.512 | 0.073 |
|  |  | 2019-2021 | -0.226 | 0.650 | 0.314 | 0.390 |

*** *P* ≤ 0.001; ** *P* ≤ 0.01; * *P* ≤ 0.05

*6. Post-hoc results of the leaf traits.*

**Table S7:** Species-wise comparison of specific leaf area (SLA), net photosynthetic light compensation point (I_c_), light-saturated net assimilation rate (A_sat_) and leaf dark respiration rate (R_d_). Differences were tested with post-hoc *t*-tests on the models in main text Table 3. Note that for I_c_, A_sat_ and R_d_, N per species is 2. Statistical significances should therefore be viewed with caution.

| Response | Species | Contrast | Estimate ± SE | df | t ratio | *P* |
| --- | --- | --- | --- | --- | --- | --- |
| SLA | Quercus | lower / upper | 1.51 ± 0.13 | 22 | 4.78 | < 0.001 *** |
| SLA | Fraxinus | lower / upper | 1.62 ± 0.18 | 22 | 4.3 | < 0.001 *** |
| SLA | Fagus | lower / upper | 1.62 ± 0.16 | 22 | 5.01 | < 0.001 *** |
| SLA | Acer | lower / upper | 1.31 ± 0.15 | 22 | 2.42 | 0.024 * |
| SLA | Carpinus | lower / upper | 1.48 ± 0.20 | 22 | 2.86 | 0.009 ** |
| SLA | Sorbus | lower / upper | 1.80 ± 0.25 | 22 | 4.30 | < 0.001 *** |
| SLA | Abies | lower / upper | 1.40 ± 0.16 | 22 | 2.99 | 0.007 ** |
| SLA | Picea | lower / upper | 1.27 ± 0.11 | 22 | 2.74 | 0.012 * |
| SLA | Pinus | lower / upper | 1.00 ± 0.10 | 22 | 0.00 | 0.997 |
| Ic | Quercus | lower - upper | -17.14 ± 11.07 | 16 | -1.55 | 0.141 |
| Ic | Fraxinus | lower - upper | -11.19 ± 11.07 | 16 | -1.01 | 0.327 |
| Ic | Fagus | lower - upper | -16.83 ± 11.07 | 16 | -1.52 | 0.148 |
| Ic | Acer | lower - upper | -18.60 ± 11.07 | 16 | -1.68 | 0.112 |
| Ic | Carpinus | lower - upper | -10.11 ± 11.07 | 16 | -0.91 | 0.375 |
| Ic | Sorbus | lower - upper | -13.41 ± 11.07 | 16 | -1.21 | 0.243 |
| Ic | Abies | lower - upper | -63.99 ± 11.07 | 16 | -5.78 | < 0.001 *** |
| Ic | Picea | lower - upper | -37.83 ± 11.07 | 16 | -3.42 | 0.004 ** |
| Ic | Pinus | lower - upper | 3.71 ± 11.07 | 16 | 0.33 | 0.742 |
| Asat | Quercus | lower - upper | -8.43 ± 1.88 | 16 | -4.48 | < 0.001 *** |
| Asat | Fraxinus | lower - upper | 0.42 ± 1.88 | 16 | 0.22 | 0.828 |
| Asat | Fagus | lower - upper | -5.31 ± 1.88 | 16 | -2.82 | 0.012 * |
| Asat | Acer | lower - upper | 0.62 ± 1.88 | 16 | 0.33 | 0.747 |
| Asat | Carpinus | lower - upper | -2.80 ± 1.88 | 16 | -1.49 | 0.157 |
| Asat | Sorbus | lower - upper | -3.14 ± 1.88 | 16 | -1.67 | 0.115 |
| Asat | Abies | lower - upper | -1.02 ± 1.88 | 16 | -0.54 | 0.594 |
| Asat | Picea | lower - upper | 0.00 ± 1.88 | 16 | 0.00 | 0.999 |
| Asat | Pinus | lower - upper | -4.36 ± 1.88 | 16 | -2.32 | 0.034 * |
| Rd | Quercus | lower - upper | 0.65 ± 0.32 | 16 | 2.04 | 0.058 |
| Rd | Fraxinus | lower - upper | 0.52 ± 0.32 | 16 | 1.62 | 0.124 |
| Rd | Fagus | lower - upper | 0.37 ± 0.32 | 16 | 1.15 | 0.268 |
| Rd | Acer | lower - upper | 0.95 ± 0.32 | 16 | 2.97 | 0.009 ** |
| Rd | Carpinus | lower - upper | 0.41 ± 0.32 | 16 | 1.29 | 0.214 |
| Rd | Sorbus | lower - upper | 0.60 ± 0.32 | 16 | 1.90 | 0.076 |
| Rd | Abies | lower - upper | 0.63 ± 0.32 | 16 | 1.97 | 0.066 |
| Rd | Picea | lower - upper | 0.64 ± 0.32 | 16 | 2.01 | 0.061 |
| Rd | Pinus | lower - upper | 0.49 ± 0.32 | 16 | 1.54 | 0.144 |

*** *P* ≤ 0.001; ** *P* ≤ 0.01; * *P* ≤ 0.05

*7. NSC concentrations in leaves of broadleaved species.*

**

**Fig. S4:** NSC concentrations in leaves of broadleaved species measured in early summer 2020. Starch (left panel) and sugar (right panel) concentrations in leaves from upper (red) and lower (blue) crown positions for each of the six broadleaved species. Samples were taken on 4. May (*Carpinus*), 14. May (*Quercus*, *Fraxinus*, *Sorbus*), 28. May (*Fagus*) and 5. June (*Acer*), corresponding to sampling dates shortly after leaves of the respective species had fully developed leaves. Concentrations did overall not differ between crown positions (Table S8), although post-hoc t tests showed small but significant differences in starch concentrations of *Fraxinus* leaves (P = 0.048) and sugar concentrations of *Carpinus* (P = 0.017).

**Table S8:** Analysis of Deviance table (type II Wald χ^2^ tests) of starch and sugar concentrations in the leaves of broadleaved species, measured in early summer 2020. Linear mixed effects models of both Starch and Sugar contained a tree ID as random intercept in addition to the fixed effects shown below.

| Response | Variable | χ^2^ | df | *P* |
| --- | --- | --- | --- | --- |
| Starch | Crown position | 0.50 | 1 | 0.481 ^ns^ |
|  | Species | 35.53 | 5 | <0.001 *** |
|  | Crown position:Species | 9.56 | 5 | 0.089 ^ns^ |
| Sugar | Crown position | 1.29 | 1 | 0.255 ^ns^ |
|  | Species | 112.24 | 5 | <0.001 *** |
|  | Crown position:Species | 9.37 | 5 | 0.095 ^ns^ |

*** *P* ≤ 0.001; ** *P* ≤ 0.01; * *P* ≤ 0.05; ns *P* > 0.05
